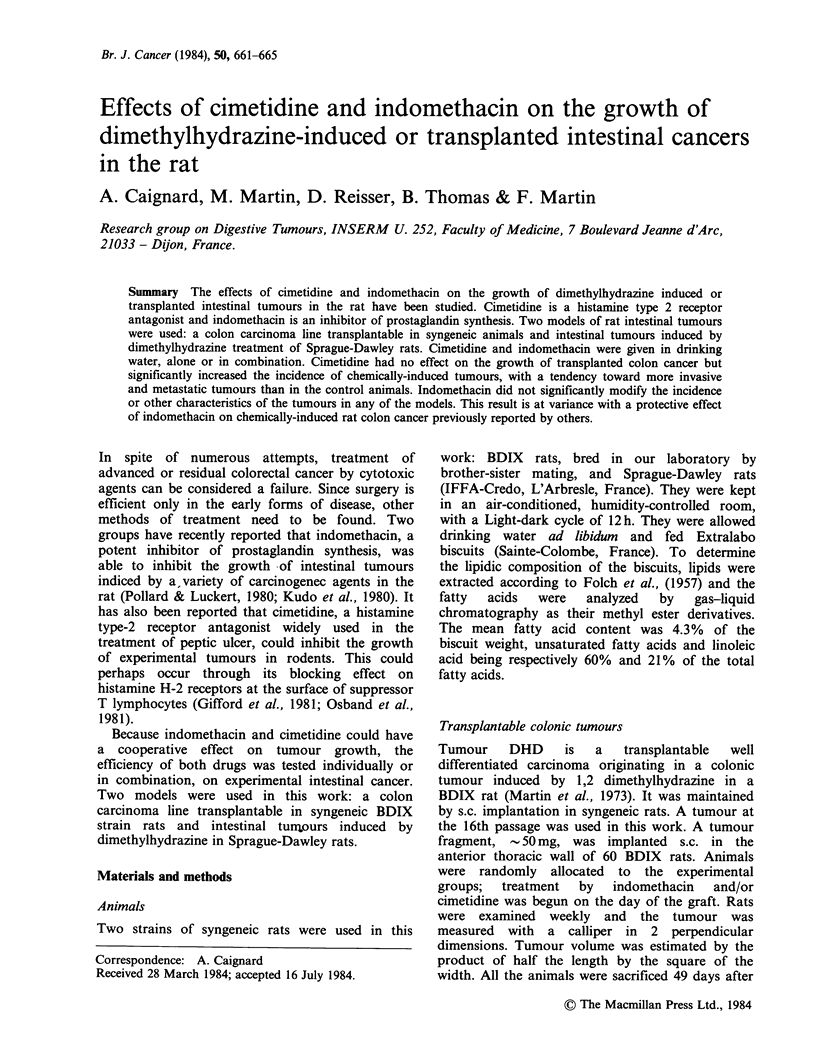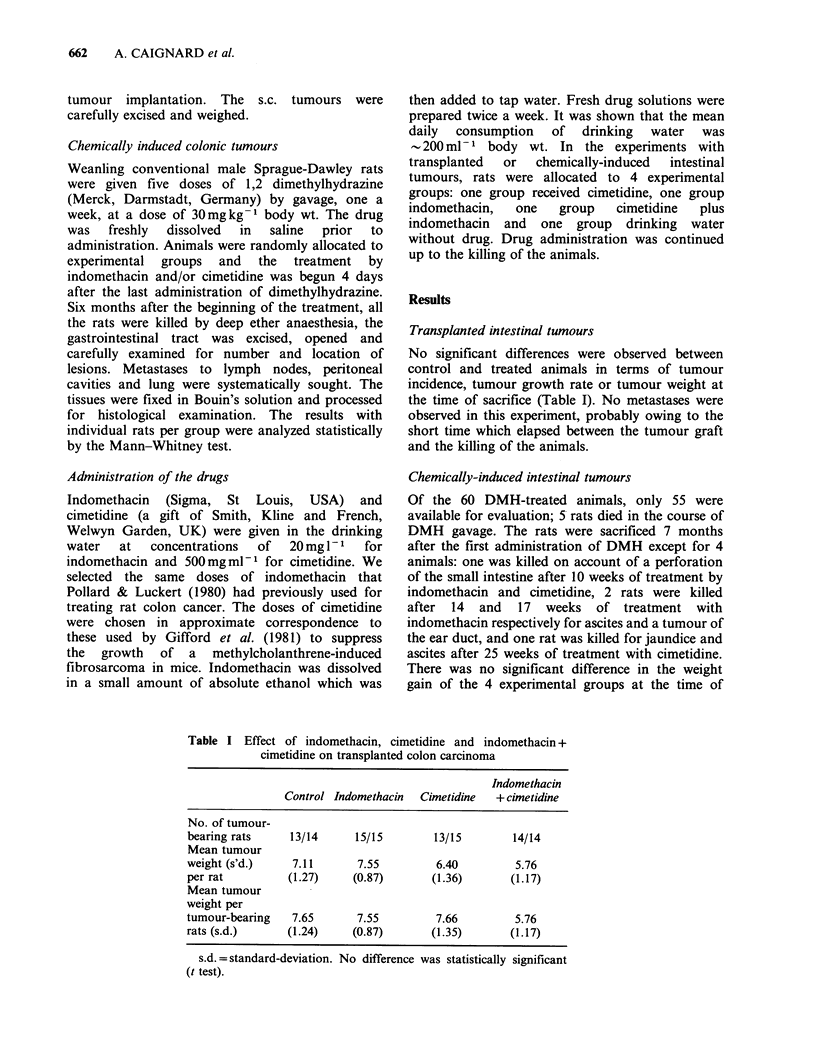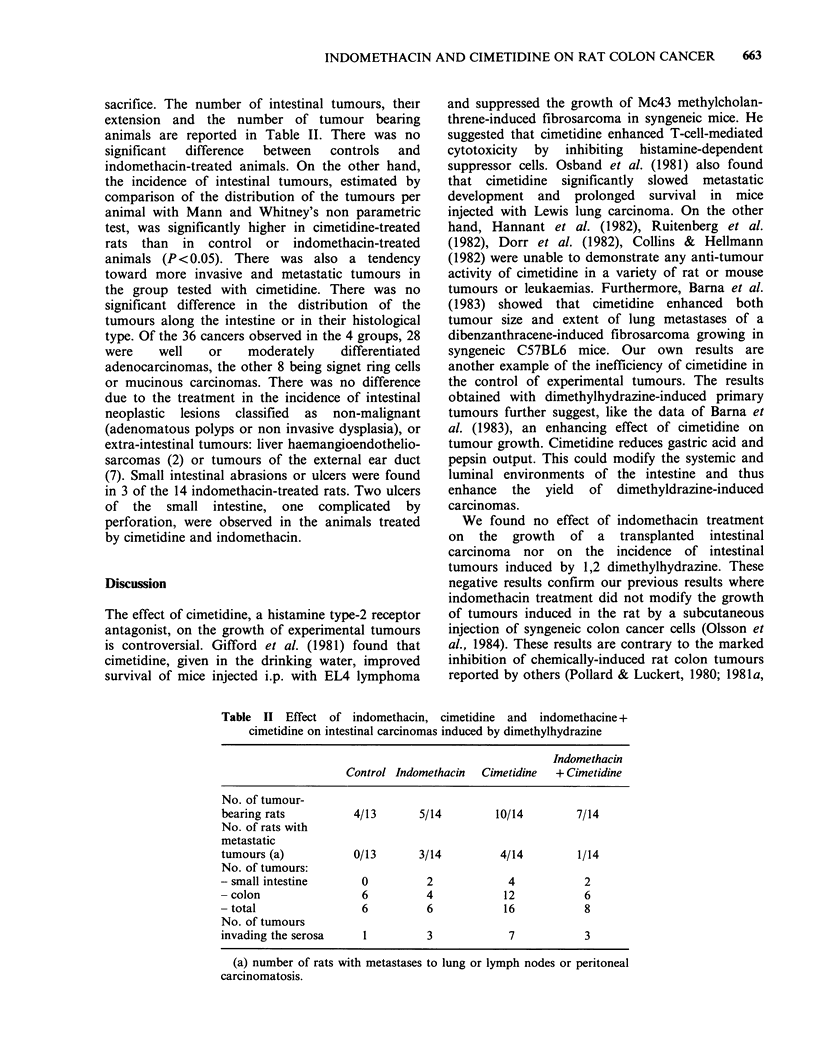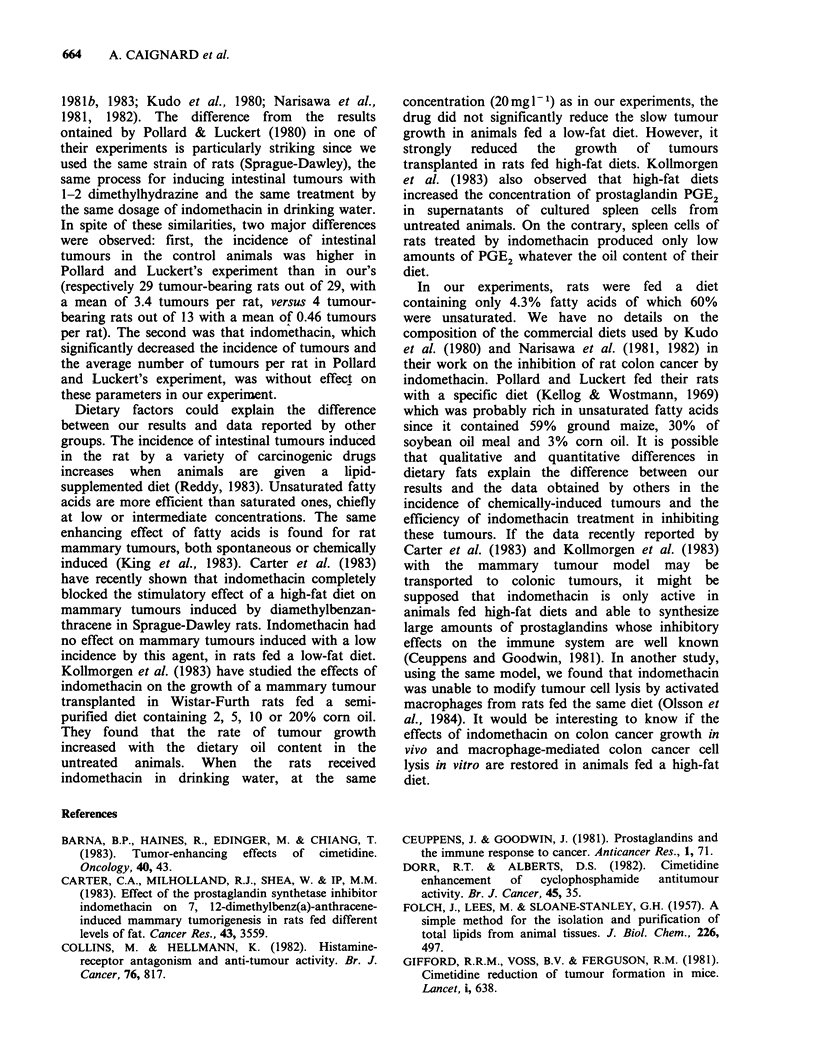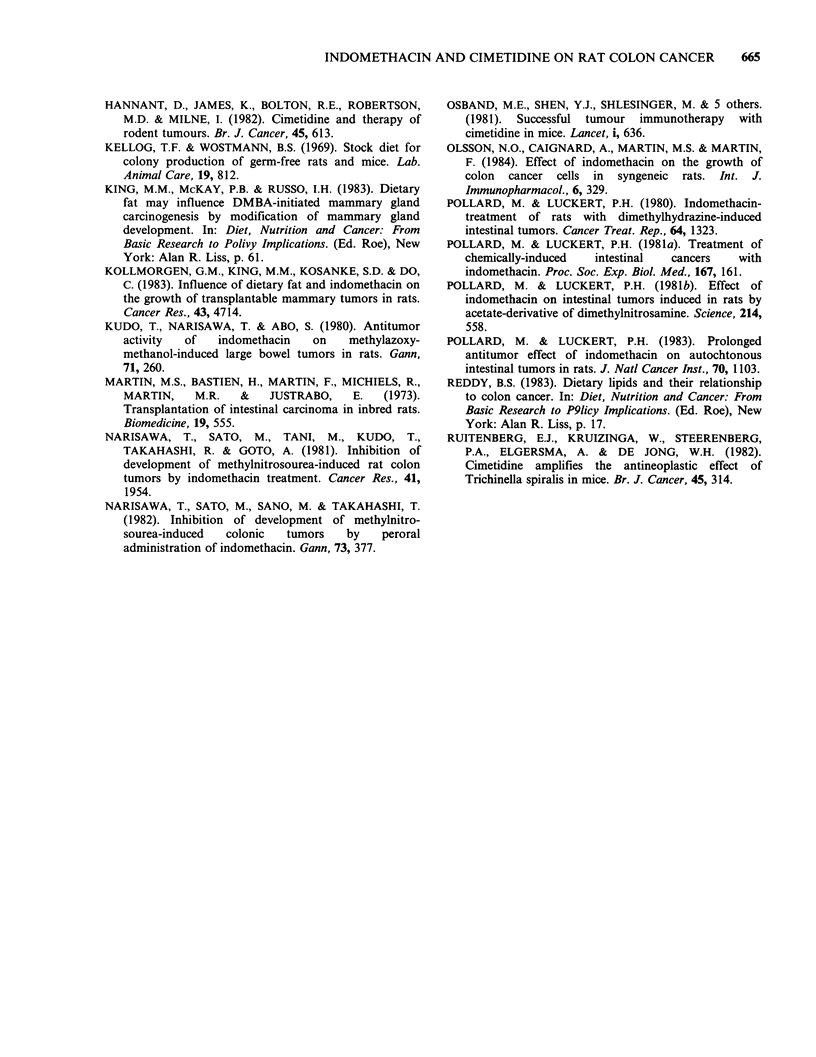# Effects of cimetidine and indomethacin on the growth of dimethylhydrazine-induced or transplanted intestinal cancers in the rat.

**DOI:** 10.1038/bjc.1984.233

**Published:** 1984-11

**Authors:** A. Caignard, M. Martin, D. Reisser, B. Thomas, F. Martin

## Abstract

The effects of cimetidine and indomethacin on the growth of dimethylhydrazine induced or transplanted intestinal tumours in the rat have been studied. Cimetidine is a histamine type 2 receptor antagonist and indomethacin is an inhibitor of prostaglandin synthesis. Two models of rat intestinal tumours were used: a colon carcinoma line transplantable in syngeneic animals and intestinal tumours induced by dimethylhydrazine treatment of Sprague-Dawley rats. Cimetidine and indomethacin were given in drinking water, alone or in combination. Cimetidine had no effect on the growth of transplanted colon cancer but significantly increased the incidence of chemically-induced tumours, with a tendency toward more invasive and metastatic tumours than in the control animals. Indomethacin did not significantly modify the incidence or other characteristics of the tumours in any of the models. This result is at variance with a protective effect of indomethacin on chemically-induced rat colon cancer previously reported by others.


*Br. J. Cancer* (1984), **50**, 661–665

### Effects of cimetidine and indomethacin on the growth of dimethylhydrazine-induced or transplanted intestinal cancers in the rat

A. Caignard, M. Martin, D. Reisser, B. Thomas & F. Martin

*Research group on Digestive Tumours, INSERM U. 252, Faculty of Medicine, 7 Boulevard Jeanne d'Arc, 21033 – Dijon, France.*

**Summary** The effects of cimetidine and indomethacin on the growth of dimethylhydrazine induced or transplanted intestinal tumours in the rat have been studied. Cimetidine is a histamine type 2 receptor antagonist and indomethacin is an inhibitor of prostaglandin synthesis. Two models of rat intestinal tumours were used: a colon carcinoma line transplantable in syngeneic animals and intestinal tumours induced by dimethylhydrazine treatment of Sprague-Dawley rats. Cimetidine and indomethacin were given in drinking water, alone or in combination. Cimetidine had no effect on the growth of transplanted colon cancer but significantly increased the incidence of chemically-induced tumours, with a tendency toward more invasive and metastatic tumours than in the control animals. Indomethacin did not significantly modify the incidence or other characteristics of the tumours in any of the models. This result is at variance with a protective effect of indomethacin on chemically-induced rat colon cancer previously reported by others.

In spite of numerous attempts, treatment of advanced or residual colorectal cancer by cytotoxic agents can be considered a failure. Since surgery is efficient only in the early forms of disease, other methods of treatment need to be found. Two groups have recently reported that indomethacin, a potent inhibitor of prostaglandin synthesis, was able to inhibit the growth of intestinal tumours indiced by a, variety of carcinogenec agents in the rat (Pollard & Luckert, 1980; Kudo *et al.*, 1980). It has also been reported that cimetidine, a histamine type-2 receptor antagonist widely used in the treatment of peptic ulcer, could inhibit the growth of experimental tumours in rodents. This could perhaps occur through its blocking effect on histamine H-2 receptors at the surface of suppressor T lymphocytes (Gifford *et al.*, 1981; Osband *et al.*, 1981).

Because indomethacin and cimetidine could have a cooperative effect on tumour growth, the efficiency of both drugs was tested individually or in combination, on experimental intestinal cancer. Two models were used in this work: a colon carcinoma line transplantable in syngeneic BDIX strain rats and intestinal tumours induced by dimethylhydrazine in Sprague-Dawley rats.

### Materials and methods

### *Animals*

Two strains of syngeneic rats were used in this work: BDIX rats, bred in our laboratory by brother-sister mating, and Sprague-Dawley rats (IFFA-Credo, L'Arbresle, France). They were kept in an air-conditioned, humidity-controlled room, with a Light-dark cycle of 12 h. They were allowed drinking water *ad libidum* and fed Extralabo biscuits (Sainte-Colombe, France). To determine the lipidic composition of the biscuits, lipids were extracted according to Folch *et al.*, (1957) and the fatty acids were analyzed by gas–liquid chromatography as their methyl ester derivatives. The mean fatty acid content was 4.3% of the biscuit weight, unsaturated fatty acids and linoleic acid being respectively 60% and 21% of the total fatty acids.

### *Transplantable colonic tumours*

Tumour DHD is a transplantable well differentiated carcinoma originating in a colonic tumour induced by 1,2 dimethylhydrazine in a BDIX rat (Martin *et al.*, 1973). It was maintained by s.c. implantation in syngeneic rats. A tumour at the 16th passage was used in this work. A tumour fragment, ~50 mg, was implanted s.c. in the anterior thoracic wall of 60 BDIX rats. Animals were randomly allocated to the experimental groups; treatment by indomethacin and/or cimetidine was begun on the day of the graft. Rats were examined weekly and the tumour was measured with a calliper in 2 perpendicular dimensions. Tumour volume was estimated by the product of half the length by the square of the width. All the animals were sacrificed 49 days after

Correspondence: A. Caignard
Received 28 March 1984; accepted 16 July 1984.

© The Macmillan Press Ltd., 1984

tumour implantation. The s.c. tumours were carefully excised and weighed.

*Chemically induced colonic tumours*

Weanling conventional male Sprague-Dawley rats were given five doses of 1,2 dimethylhydrazine (Merck, Darmstadt, Germany) by gavage, one a week, at a dose of $30\,mg\,kg^{-1}$ body wt. The drug was freshly dissolved in saline prior to administration. Animals were randomly allocated to experimental groups and the treatment by indomethacin and/or cimetidine was begun 4 days after the last administration of dimethylhydrazine. Six months after the beginning of the treatment, all the rats were killed by deep ether anaesthesia, the gastrointestinal tract was excised, opened and carefully examined for number and location of lesions. Metastases to lymph nodes, peritoneal cavities and lung were systematically sought. The tissues were fixed in Bouin's solution and processed for histological examination. The results with individual rats per group were analyzed statistically by the Mann–Whitney test.

*Administration of the drugs*

Indomethacin (Sigma, St Louis, USA) and cimetidine (a gift of Smith, Kline and French, Welwyn Garden, UK) were given in the drinking water at concentrations of $20\,mg\,l^{-1}$ for indomethacin and $500\,mg\,ml^{-1}$ for cimetidine. We selected the same doses of indomethacin that Pollard & Luckert (1980) had previously used for treating rat colon cancer. The doses of cimetidine were chosen in approximate correspondence to these used by Gifford *et al.* (1981) to suppress the growth of a methylcholanthrene-induced fibrosarcoma in mice. Indomethacin was dissolved in a small amount of absolute ethanol which was then added to tap water. Fresh drug solutions were prepared twice a week. It was shown that the mean daily consumption of drinking water was $\sim 200\,ml^{-1}$ body wt. In the experiments with transplanted or chemically-induced intestinal tumours, rats were allocated to 4 experimental groups: one group received cimetidine, one group indomethacin, one group cimetidine plus indomethacin and one group drinking water without drug. Drug administration was continued up to the killing of the animals.

### Results

*Transplanted intestinal tumours*

No significant differences were observed between control and treated animals in terms of tumour incidence, tumour growth rate or tumour weight at the time of sacrifice (Table I). No metastases were observed in this experiment, probably owing to the short time which elapsed between the tumour graft and the killing of the animals.

*Chemically-induced intestinal tumours*

Of the 60 DMH-treated animals, only 55 were available for evaluation; 5 rats died in the course of DMH gavage. The rats were sacrificed 7 months after the first administration of DMH except for 4 animals: one was killed on account of a perforation of the small intestine after 10 weeks of treatment by indomethacin and cimetidine, 2 rats were killed after 14 and 17 weeks of treatment with indomethacin respectively for ascites and a tumour of the ear duct, and one rat was killed for jaundice and ascites after 25 weeks of treatment with cimetidine. There was no significant difference in the weight gain of the 4 experimental groups at the time of

**Table I** Effect of indomethacin, cimetidine and indomethacin + cimetidine on transplanted colon carcinoma

| | *Control* | *Indomethacin* | *Cimetidine* | *Indomethacin + cimetidine* |
|---|---|---|---|---|
| No. of tumour-bearing rats | 13/14 | 15/15 | 13/15 | 14/14 |
| Mean tumour weight (s'd.) per rat | 7.11 (1.27) | 7.55 (0.87) | 6.40 (1.36) | 5.76 (1.17) |
| Mean tumour weight per tumour-bearing rats (s.d.) | 7.65 (1.24) | 7.55 (0.87) | 7.66 (1.35) | 5.76 (1.17) |

s.d. = standard-deviation. No difference was statistically significant (*t* test).

sacrifice. The number of intestinal tumours, their extension and the number of tumour bearing animals are reported in Table II. There was no significant difference between controls and indomethacin-treated animals. On the other hand, the incidence of intestinal tumours, estimated by comparison of the distribution of the tumours per animal with Mann and Whitney's non parametric test, was significantly higher in cimetidine-treated rats than in control or indomethacin-treated animals ($P<0.05$). There was also a tendency toward more invasive and metastatic tumours in the group tested with cimetidine. There was no significant difference in the distribution of the tumours along the intestine or in their histological type. Of the 36 cancers observed in the 4 groups, 28 were well or moderately differentiated adenocarcinomas, the other 8 being signet ring cells or mucinous carcinomas. There was no difference due to the treatment in the incidence of intestinal neoplastic lesions classified as non-malignant (adenomatous polyps or non invasive dysplasia), or extra-intestinal tumours: liver haemangioendotheliosarcomas (2) or tumours of the external ear duct (7). Small intestinal abrasions or ulcers were found in 3 of the 14 indomethacin-treated rats. Two ulcers of the small intestine, one complicated by perforation, were observed in the animals treated by cimetidine and indomethacin.

### Discussion

The effect of cimetidine, a histamine type-2 receptor antagonist, on the growth of experimental tumours is controversial. Gifford *et al.* (1981) found that cimetidine, given in the drinking water, improved survival of mice injected i.p. with EL4 lymphoma and suppressed the growth of Mc43 methylcholanthrene-induced fibrosarcoma in syngeneic mice. He suggested that cimetidine enhanced T-cell-mediated cytotoxicity by inhibiting histamine-dependent suppressor cells. Osband *et al.* (1981) also found that cimetidine significantly slowed metastatic development and prolonged survival in mice injected with Lewis lung carcinoma. On the other hand, Hannant *et al.* (1982), Ruitenberg *et al.* (1982), Dorr *et al.* (1982), Collins & Hellmann (1982) were unable to demonstrate any anti-tumour activity of cimetidine in a variety of rat or mouse tumours or leukaemias. Furthermore, Barna *et al.* (1983) showed that cimetidine enhanced both tumour size and extent of lung metastases of a dibenzanthracene-induced fibrosarcoma growing in syngeneic C57BL6 mice. Our own results are another example of the inefficiency of cimetidine in the control of experimental tumours. The results obtained with dimethylhydrazine-induced primary tumours further suggest, like the data of Barna *et al.* (1983), an enhancing effect of cimetidine on tumour growth. Cimetidine reduces gastric acid and pepsin output. This could modify the systemic and luminal environments of the intestine and thus enhance the yield of dimethyldrazine-induced carcinomas.

We found no effect of indomethacin treatment on the growth of a transplanted intestinal carcinoma nor on the incidence of intestinal tumours induced by 1,2 dimethylhydrazine. These negative results confirm our previous results where indomethacin treatment did not modify the growth of tumours induced in the rat by a subcutaneous injection of syngeneic colon cancer cells (Olsson *et al.*, 1984). These results are contrary to the marked inhibition of chemically-induced rat colon tumours reported by others (Pollard & Luckert, 1980; 1981*a*,

**Table II** Effect of indomethacin, cimetidine and indomethacine+cimetidine on intestinal carcinomas induced by dimethylhydrazine

| | *Control* | *Indomethacin* | *Cimetidine* | *Indomethacin + Cimetidine* |
|---|---|---|---|---|
| No. of tumour-bearing rats | 4/13 | 5/14 | 10/14 | 7/14 |
| No. of rats with metastatic tumours (a) | 0/13 | 3/14 | 4/14 | 1/14 |
| No. of tumours: | | | | |
| – small intestine | 0 | 2 | 4 | 2 |
| – colon | 6 | 4 | 12 | 6 |
| – total | 6 | 6 | 16 | 8 |
| No. of tumours invading the serosa | 1 | 3 | 7 | 3 |

(a) number of rats with metastases to lung or lymph nodes or peritoneal carcinomatosis.

1981*b*, 1983; Kudo *et al.*, 1980; Narisawa *et al.*, 1981, 1982). The difference from the results ontained by Pollard & Luckert (1980) in one of their experiments is particularly striking since we used the same strain of rats (Sprague-Dawley), the same process for inducing intestinal tumours with 1–2 dimethylhydrazine and the same treatment by the same dosage of indomethacin in drinking water. In spite of these similarities, two major differences were observed: first, the incidence of intestinal tumours in the control animals was higher in Pollard and Luckert's experiment than in our's (respectively 29 tumour-bearing rats out of 29, with a mean of 3.4 tumours per rat, *versus* 4 tumour-bearing rats out of 13 with a mean of 0.46 tumours per rat). The second was that indomethacin, which significantly decreased the incidence of tumours and the average number of tumours per rat in Pollard and Luckert's experiment, was without effect on these parameters in our experiment.

Dietary factors could explain the difference between our results and data reported by other groups. The incidence of intestinal tumours induced in the rat by a variety of carcinogenic drugs increases when animals are given a lipid-supplemented diet (Reddy, 1983). Unsaturated fatty acids are more efficient than saturated ones, chiefly at low or intermediate concentrations. The same enhancing effect of fatty acids is found for rat mammary tumours, both spontaneous or chemically induced (King *et al.*, 1983). Carter *et al.* (1983) have recently shown that indomethacin completely blocked the stimulatory effect of a high-fat diet on mammary tumours induced by diamethylbenzanthracene in Sprague-Dawley rats. Indomethacin had no effect on mammary tumours induced with a low incidence by this agent, in rats fed a low-fat diet. Kollmorgen *et al.* (1983) have studied the effects of indomethacin on the growth of a mammary tumour transplanted in Wistar-Furth rats fed a semi-purified diet containing 2, 5, 10 or 20% corn oil. They found that the rate of tumour growth increased with the dietary oil content in the untreated animals. When the rats received indomethacin in drinking water, at the same concentration ($20\,mg\,l^{-1}$) as in our experiments, the drug did not significantly reduce the slow tumour growth in animals fed a low-fat diet. However, it strongly reduced the growth of tumours transplanted in rats fed high-fat diets. Kollmorgen *et al.* (1983) also observed that high-fat diets increased the concentration of prostaglandin $PGE_2$ in supernatants of cultured spleen cells from untreated animals. On the contrary, spleen cells of rats treated by indomethacin produced only low amounts of $PGE_2$ whatever the oil content of their diet.

In our experiments, rats were fed a diet containing only 4.3% fatty acids of which 60% were unsaturated. We have no details on the composition of the commercial diets used by Kudo *et al.* (1980) and Narisawa *et al.* (1981, 1982) in their work on the inhibition of rat colon cancer by indomethacin. Pollard and Luckert fed their rats with a specific diet (Kellog & Wostmann, 1969) which was probably rich in unsaturated fatty acids since it contained 59% ground maize, 30% of soybean oil meal and 3% corn oil. It is possible that qualitative and quantitative differences in dietary fats explain the difference between our results and the data obtained by others in the incidence of chemically-induced tumours and the efficiency of indomethacin treatment in inhibiting these tumours. If the data recently reported by Carter *et al.* (1983) and Kollmorgen *et al.* (1983) with the mammary tumour model may be transported to colonic tumours, it might be supposed that indomethacin is only active in animals fed high-fat diets and able to synthesize large amounts of prostaglandins whose inhibitory effects on the immune system are well known (Ceuppens and Goodwin, 1981). In another study, using the same model, we found that indomethacin was unable to modify tumour cell lysis by activated macrophages from rats fed the same diet (Olsson *et al.*, 1984). It would be interesting to know if the effects of indomethacin on colon cancer growth *in vivo* and macrophage-mediated colon cancer cell lysis *in vitro* are restored in animals fed a high-fat diet.

### References

BARNA, B.P., HAINES, R., EDINGER, M. & CHIANG, T. (1983). Tumor-enhancing effects of cimetidine. *Oncology*, **40**, 43.

CARTER, C.A., MILHOLLAND, R.J., SHEA, W. & IP, M.M. (1983). Effect of the prostaglandin synthetase inhibitor indomethacin on 7, 12-dimethylbenz(a)-anthracene-induced mammary tumorigenesis in rats fed different levels of fat. *Cancer Res.*, **43**, 3559.

COLLINS, M. & HELLMANN, K. (1982). Histamine-receptor antagonism and anti-tumour activity. *Br. J. Cancer*, **76**, 817.

CEUPPENS, J. & GOODWIN, J. (1981). Prostaglandins and the immune response to cancer. *Anticancer Res.*, **1**, 71.

DORR, R.T. & ALBERTS, D.S. (1982). Cimetidine enhancement of cyclophosphamide antitumour activity. *Br. J. Cancer*, **45**, 35.

FOLCH, J., LEES, M. & SLOANE-STANLEY, G.H. (1957). A simple method for the isolation and purification of total lipids from animal tissues. *J. Biol. Chem.*, **226**, 497.

GIFFORD, R.R.M., VOSS, B.V. & FERGUSON, R.M. (1981). Cimetidine reduction of tumour formation in mice. *Lancet*, **i**, 638.

HANNANT, D., JAMES, K., BOLTON, R.E., ROBERTSON, M.D. & MILNE, I. (1982). Cimetidine and therapy of rodent tumours. *Br. J. Cancer*, **45**, 613.

KELLOG, T.F. & WOSTMANN, B.S. (1969). Stock diet for colony production of germ-free rats and mice. *Lab. Animal Care*, **19**, 812.

KING, M.M., McKAY, P.B. & RUSSO, I.H. (1983). Dietary fat may influence DMBA-initiated mammary gland carcinogenesis by modification of mammary gland development. In: *Diet, Nutrition and Cancer: From Basic Research to Polivy Implications*. (Ed. Roe), New York: Alan R. Liss, p. 61.

KOLLMORGEN, G.M., KING, M.M., KOSANKE, S.D. & DO, C. (1983). Influence of dietary fat and indomethacin on the growth of transplantable mammary tumors in rats. *Cancer Res.*, **43**, 4714.

KUDO, T., NARISAWA, T. & ABO, S. (1980). Antitumor activity of indomethacin on methylazoxy-methanol-induced large bowel tumors in rats. *Gann*, **71**, 260.

MARTIN, M.S., BASTIEN, H., MARTIN, F., MICHIELS, R., MARTIN, M.R. & JUSTRABO, E. (1973). Transplantation of intestinal carcinoma in inbred rats. *Biomedicine*, **19**, 555.

NARISAWA, T., SATO, M., TANI, M., KUDO, T., TAKAHASHI, R. & GOTO, A. (1981). Inhibition of development of methylnitrosourea-induced rat colon tumors by indomethacin treatment. *Cancer Res.*, **41**, 1954.

NARISAWA, T., SATO, M., SANO, M. & TAKAHASHI, T. (1982). Inhibition of development of methylnitrosourea-induced colonic tumors by peroral administration of indomethacin. *Gann*, **73**, 377.

OSBAND, M.E., SHEN, Y.J., SHLESINGER, M. & 5 others. (1981). Successful tumour immunotherapy with cimetidine in mice. *Lancet*, **i**, 636.

OLSSON, N.O., CAIGNARD, A., MARTIN, M.S. & MARTIN, F. (1984). Effect of indomethacin on the growth of colon cancer cells in syngeneic rats. *Int. J. Immunopharmacol.*, **6**, 329.

POLLARD, M. & LUCKERT, P.H. (1980). Indomethacin-treatment of rats with dimethylhydrazine-induced intestinal tumors. *Cancer Treat. Rep.*, **64**, 1323.

POLLARD, M. & LUCKERT, P.H. (1981*a*). Treatment of chemically-induced intestinal cancers with indomethacin. *Proc. Soc. Exp. Biol. Med.*, **167**, 161.

POLLARD, M. & LUCKERT, P.H. (1981*b*). Effect of indomethacin on intestinal tumors induced in rats by acetate-derivative of dimethylnitrosamine. *Science*, **214**, 558.

POLLARD, M. & LUCKERT, P.H. (1983). Prolonged antitumor effect of indomethacin on autochtonous intestinal tumors in rats. *J. Natl Cancer Inst.*, **70**, 1103.

REDDY, B.S. (1983). Dietary lipids and their relationship to colon cancer. In: *Diet, Nutrition and Cancer: From Basic Research to P9licy Implications*. (Ed. Roe), New York: Alan R. Liss, p. 17.

RUITENBERG, E.J., KRUIZINGA, W., STEERENBERG, P.A., ELGERSMA, A. & DE JONG, W.H. (1982). Cimetidine amplifies the antineoplastic effect of Trichinella spiralis in mice. *Br. J. Cancer*, **45**, 314.